# Neutralization against Omicron sublineages (BA.2/BA.5/BQ.1.1/XBB/XBB.1.5) in bivalent BNT162b2-vaccinated HCWs with or without risk factors, or following BT infection with Omicron

**DOI:** 10.1038/s41598-023-44484-x

**Published:** 2023-10-13

**Authors:** Masayuki Amano, Sachiko Otsu, Yukari Uemura, Yasuko Ichikawa, Shota Matsumoto, Nobuyo Higashi-Kuwata, Shuzo Matsushita, Shinya Shimada, Hiroaki Mitsuya

**Affiliations:** 1 Department of Clinical Retrovirology, Joint Research Center for Human Retrovirus Infection, Kumamoto University, 2-2-1 Honjo, Cyuou-ku, Kumamoto, 860-0811 Japan; 2 Department of Data Sciences, Center for Clinical Sciences, National Center for Global Health and Medicine (NCGM), Tokyo, Japan; 3 Kumamoto General Hospital, Japan Community Healthcare Organization (JCHO), Kumamoto, Japan; 4 Department of Refractory Viral Diseases, NCGM Research Institute, Tokyo, Japan

**Keywords:** Infectious diseases, Infectious diseases, Vaccines

## Abstract

SARS-CoV-2-BA.4/5-adapted-bivalent-BNT162b2-vaccine (^bv^BNT), developed in response to the recent emergence of immune-evasive Omicron-variants, has been given to individuals who completed at least 2-doses of the monovalent-BNT162b2-vaccine (^mv^BNT). In the present cohort study, we evaluated neutralization-titers (NT_50_s) against Wuhan-strain (SCoV2^Wuhan^) and Omicron-sublineages including BA.2/BA.5/BQ.1.1/XBB/XBB.1.5, and vaccine-elicited S1-binding-IgG in sera from participants-vaccinated with 5th-^bv^BNT following 4th-^mv^BNT. The 5th-^bv^BNT-dose elicited good protective-activity against SCoV2^Wuhan^ with geometric-mean (gMean)-NT_50_ of 1966–2091, higher than the peak-values post-4th-^mv^BNT with no statistical significance, and favorable neutralization-activity against not only BA.5 but also BA.2, with ~ 3.2-/~ 2.2-fold greater gMean-NT_50_ compared to the peak-values post-4th-^mv^BNT-dose, in participants with or without risk factors. However, neutralization-activity of sera post-5th-^bv^BNT-dose was low against BQ.1.1/XBB/XBB.1.5. Interestingly, participants receiving ^bv^BNT following breakthrough (BT) infection during Omicron-wave had significantly enhanced neutralization-activity against SCoV2^Wuhan^/BA.2/BA.5 with ~ 4.6-/~ 6.3-/~ 8.1-fold greater gMean-NT_50_, respectively, compared to uninfected participants receiving ^bv^BNT. Sera from BT-infected-participants receiving ^bv^BNT had enhanced neutralization-activity against BQ.1.1/XBB/XBB.1.5 by ~ 3.8-fold compared to those from the same participants post-4th-^mv^BNT-dose, and had enhanced gMean-NT_50_ ~ 5.4-fold greater compared to those of uninfected-participants’ sera post-^bv^BNT. These results suggest that repeated stimulation brought about by exposure to BA.5’s-Spike elicit favorable cross-neutralization-activity against various SARS-CoV-2-variants.

## Introduction

Since the emergence of coronavirus disease 2019 (COVID-19) caused by severe acute respiratory syndrome coronavirus 2 (SARS-CoV-2) infection in Wuhan, COVID-19 rapidly spread worldwide. At present, 754 million SARS-CoV-2-confirmed cases and more than 6.8 million of deaths by COVID-19 have been reported as of February 5, 2023, globally^1–4^.

From the initial stage of the global pandemic, massive efforts were made toward development of novel vaccines against SARS-CoV-2 around the world^5–7^ Currently, more than 50 vaccines have been approved by at least one country (https://covid19.trackvaccines.org/vaccines/approved/). The efficacy of vaccines against SARS-CoV-2 had been beyond expectation. As of February 2023, more than 13.3 billion of anti-SARS-CoV-2 vaccine doses have already been administered in the world^8^. Among various vaccines, two mRNA vaccines, BNT162b2 (Pfizer/BioNTech) and mRNA-1273 (Moderna), have previously shown ~ 95% efficacy in preventing symptomatic COVID-19 in early-phase of pandemic^9–11^, and these mRNA vaccines accounted for 90 and 97% of total administrated doses of COVID-19 vaccine in U.S. and European Union respectively, up to present^8^.

According to the recent emergence of immune-evasive Omicron variants, novel bivalent mRNA booster vaccines were developed by targeting the Spike protein of SARS-CoV-2^Wuhan^ and Omicron BA.4/BA.5 sublineages and have been provided to individuals who had completed at least 2 doses of monovalent COVID-19 vaccination. Although the Morbidity and Mortality Weekly Report (MMWR) described that among immunocompetent adults, who were ≥ 65 years, a bivalent booster dose provided 73% additional protection against COVID-19 hospitalization compared with monovalent mRNA vaccination only^12^, there have been multiple results reporting antibody evasion profiles of new Omicron sublineages BQ.1.1 and XBB, posing further concerns on the efficacy of anti-SARS-CoV-2 vaccines^13^.

We have continuously evaluated the neutralizing activity of sera obtained from Pfizer/BioNTech monovalent BNT162b2 (^mv^BNT)-vaccinated health care workers (HCWs) in Japan^14–18^. In the present proactive cohort study, we focused on the vaccinated participants’ sera obtained pre- and post-5th-dose of Omicron BA.4/5-adapted bivalent BNT162b2 (^bv^BNT), and determined neutralization titers (NT_50_s) of sera against wild-type Wuhan SARS-CoV-2 strain (SCoV2^Wuhan^) and Omicron sublineages, including BA.2, BA.5, BQ.1.1, XBB, and XBB.1.5, vaccine-elicited S1-binding IgG levels. All the SARS-CoV-2 strains/variants used in this study were infectious viruses, isolated from individuals at airport quarantine stations or hospitals in Japan and were not recombinant- or pseudo-viruses.

## Results

### Effects of Omicron BA.4/5-adapted BNT162b2 (^bv^BNT) vaccination in sera obtained from health care workers (HCWs) with risk factors.

Firstly, we examined SARS-CoV-2 neutralization activity of sera post-^bv^BNT booster vaccine dose, obtained from 23 out of 225 HCWs in Kumamoto General Hospital, Japan (225 individuals were initially recruited in the primary clinical study^14^), who were either of ≥ 60-years of age and/or had pre-existing diseases/risk factors (see demographic characteristics in Table 1).

**Table 1:** Demographic characteristics of the participants who received Omicron BA.5-adapted 5th-dose of BNT162b2 vaccination.

Participants (n)*	HCWs with risk factors (n = 23)	HCWs without risk factor (n = 90)	BT infected HCWs (n = 30)
Age			
20–29 y.o	0 (0%)	15 (21–28, 16.7%)	2 (23–27, 6.7%)
30–39 y.o	2 (33, 8.7%)	16 (30–39, 17.8%)	10 (31–39, 33.3%)
40–49 y.o	6 (40–49, 26.1%)	31 (40–49, 34.4%)	12 (40–49, 40.0%)
y.o	5 (50–58, 21.7%)	28 (50–58, 31.1%)	4 (51–58, 13.3%)
> 60 y.o	10 (60–72, 43.5%)	0 (0%)	2 (60–64, 6.7%)
Gender			
Men	7 (30.4%)	22 (24.4%)	8 (31.0%)
Women	16 (69.6%)	68 (75.5%)	22 (69.0%)

*143 of 225 health care workers participated in the study. None of the participants were in immunodeficient states or were receiving immunosuppressants or steroids. Risk factors contain age (≧ 60 y.o) and following diseases/conditions; asthma, hypertension, diabetes, malignancy, obesity, and liver disease.

**Figure 1:**
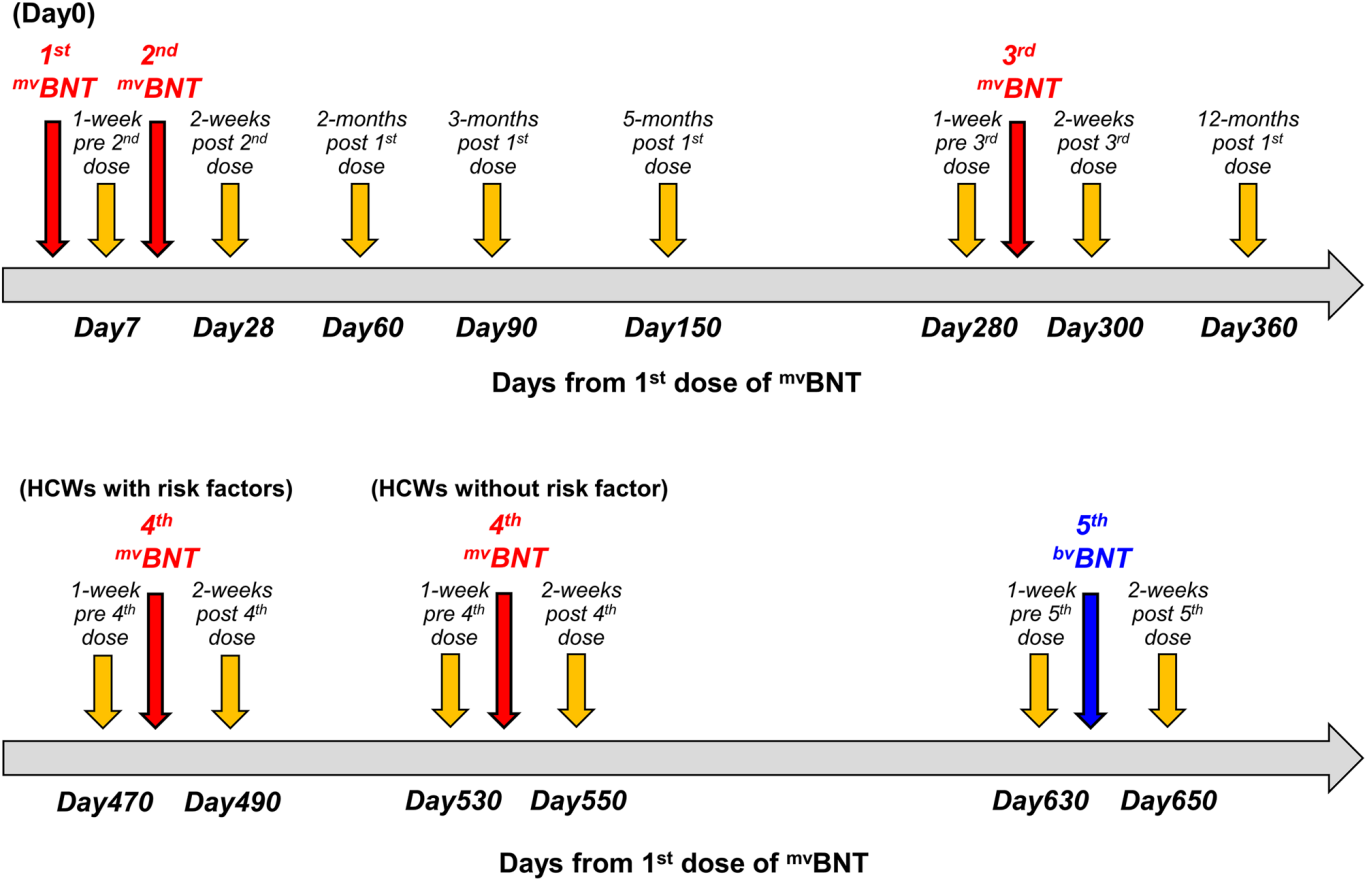
Schedule of 4-times ^mv^BNT doses and once ^bv^BNT dose in this study. Administration schedule of 4-times ^mv^BNT doses (red arrows), once ^bv^BNT dose (blue arrows), and blood collections (yellow arrows) in this study are shown. HCWs with risk factors received 4th dose of ^mv^BNT on day477, while HCWs without risk factor received it on day537. HCWs with risk factors had 11th blood collection (10-weeks post 4th dose) on day550.

The SARS-CoV-2 neutralizing activity (NT_50_) of their sera against the Wuhan strain of SARS-CoV-2 (infectious SCoV2^Wuhan^) was determined over 650 days using sera consecutively collected on (1) 1 week pre-4th-dose (day-470 [HCWs with risk factors]/-530 [HCWs without a risk factor(s)] from 1st-dose), (2) 2 weeks post-4th-dose (day-490 [HCWs with a risk factor(s)]/-550 [HCWs without a risk factor(s)]), (3) 10 weeks post-4th-dose (day-550 [HCWs with a risk factor(s) only]), (4) 1 week pre-5th-dose (^bv^BNT; day-630), and (5) 2 weeks post-5th-dose (day-650)(see Methods and Fig. 1), representing a continuation of our previous studies^14–18^. We also evaluated the profile of S1-binding IgG levels following pre-/post-3rd, 4th, and 5th-dose (Table 2). In addition, we determined NT_50_s of the same sera using VeroE6^TMPRSS^ cells against infectious Omicron BA.2, BA.5, BQ.1.1, XBB, and XBB.1.5 variant sublineages, whose emergence has been associated with the present explosive increases globally^1^. Results of samples obtained from participants whose swab PCR and/or anti-nucleocapsid-IgG in serum proved to be SARS-CoV-2 positive during this cohort study were excluded here.

**Table 2:** S1-binding IgG level of pre/post 3rd, 4th, and 5th-doses sera.

Days from initial dose	gMean S1-binding IgG (BAU/mL)		
	HCWs with risk factors (n = 23)	HCWs without risk factor (n = 90)	BT infected HCWs (n = 30)
Day280; 1 week pre 3rd-dose	135 (range; 42–537)	141 (range; 22–1137)	131 (range; 45–770)
Day300; 2 weeks post 3rd-dose	4788 (range; 1344–16,634) *[f.c. from day280 is 35.5; *p* < 0.0001]	5339 (range; 1661–23,928) [f.c. from day280 is 37.9; *p* < 0.0001]	5510 (range; 1401–15,963) [f.c. from day280 is 42.1; *p* < 0.0001]
Days470/530; 1 week pre 4th-dose	1159 (range; 240–6300)	840 (range; 99–5323)	2596 (range; 238–17,854)
Days490/550; 2 weeks post 4th-dose	6305 (range; 1611–19,236) [f.c. from day470 is 5.4; *p* < 0.0001]	6024 (range; 1912–43,502) [f.c. from day530 is 7.2; *p* < 0.0001]	11,594 (range; 1426–51,848)
Day630; 1 week pre 5th-dose	1431 (range; 389–5822)	2271 (range; 387–12,082)	8866 (range; 1891–55,214)
Day650; 2 weeks post 5th-dose	4688 (range; 846–13,334) [f.c. from day630 is 3.3; *p* < 0.0001]	4691 (range; 1313–38,324) [f.c. from day630 is 2.1; *p* < 0.0001]	14,883 (range; 3403–63,441)

Geometric mean (gMean) values of S1-binding IgG are shown. Detections of S1-binding IgG pre/post 3rd, 4th, and 5th-doses sera were conducted using the chemiluminescence enzyme immunoassay (CLEIA) platform (HISCL) manufactured by Sysmex Co. (Kobe, Japan) as previously reported^22^.

*The values at day300, days490/550 and day650 in “[]” are represented fold change (f.c.) from value of sera obtained 1-week before most recent vaccine dose at each time-point.

**Figure 2:**
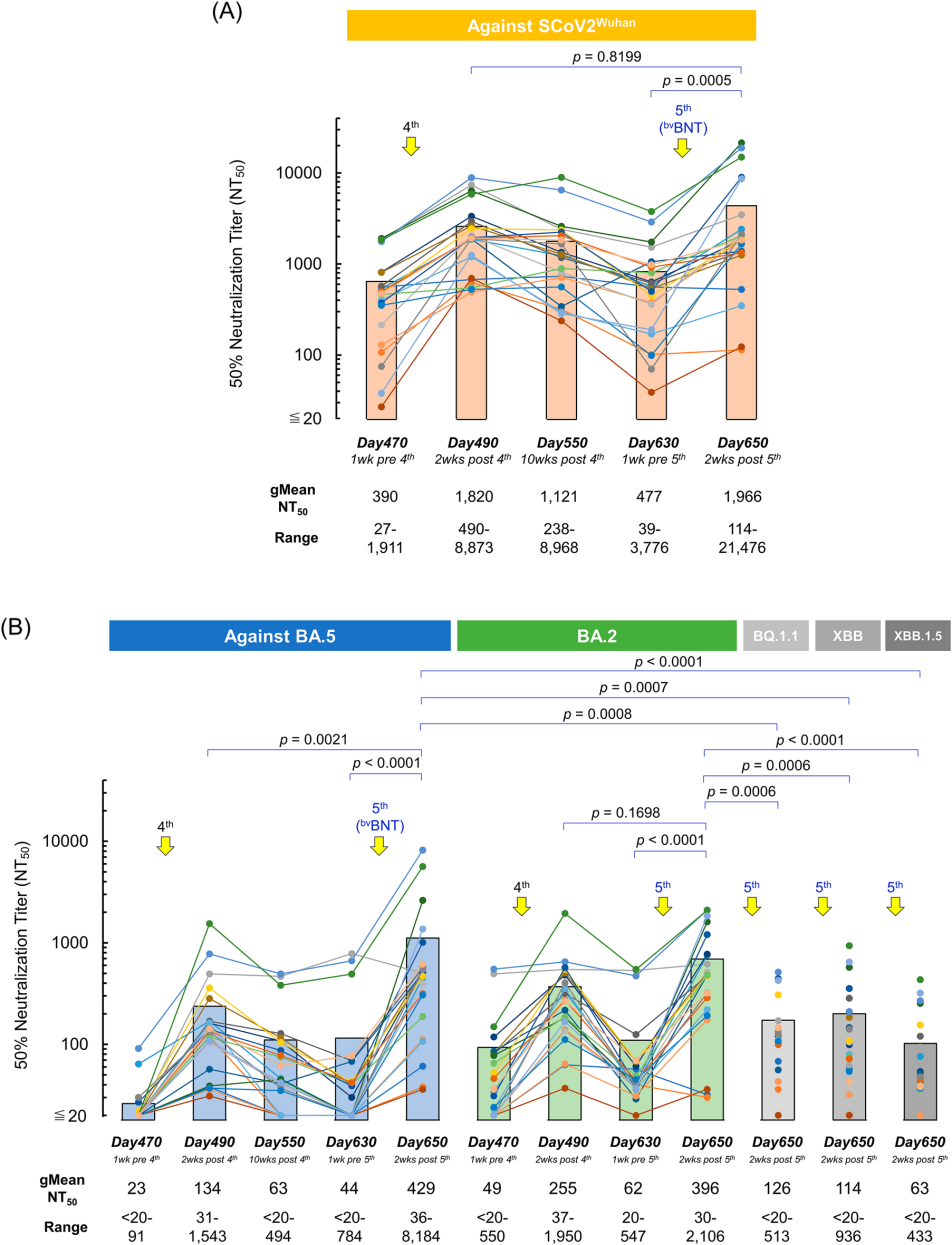
Effect of Omicron BA.4/5-adapted BNT162b2 (^bv^BNT) vaccination in sera obtained from HCWs with risk factors. Temporal changes of neutralizing activity of sera obtained from HCWs with risk factors over 650 days post-1st dose of BNT162b2 are shown. 4th monovalent (^mv^BNT) and 5th bivalent BNT162b2 (^bv^BNT) doses were administered on days 477 and 637, respectively (n = 23). (**A**) The 50% neutralization titers (NT_50_) of participants’ sera against infection by SARS-CoV-2^Wuhan^ strain (SCoV2^Wuhan^) were determined on days 470, 490, 550, 630, and 650 post-1st-dose using VeroE6^TMPRSS2^ cell-based neutralization assay. Solid circles denote NT_50_ titers of each participant’s serum and filled bars denote average NT_50_ titers of 23 participants’ sera at each time point. Geometric mean NT_50_ titers (gMean-NT_50_) and ranges of NT_50_ at each time point are shown at the bottom. (**B**) Temporal changes of neutralizing activity of participants’ sera at days 470, 490, 550, 630, and 650 post-1st dose against Omicrons BA.2, BA.5, BQ.1.1, XBB, and XBB.1.5 are shown. Solid circles denote NT_50_ titers of each participant’s serum and filled bars denote average NT_50_ titers of 23 participants’ sera at each time point. The circles and lines in same color indicate that the data were obtained from same participant’s sera.

Neutralizing activity of sera against SCoV2^Wuhan^ was seen elevated moderately on day-28 (1-week post-2nd-dose) samples (Supplemental Table S1, gMean-NT_50_ = 283), while remarkable elevations were observed in neutralizing activity in the same participants’ sera of day-300 (2-weeks post-3rd-dose), achieving gMean-NT_50_ of 2009 (Supplemental Table S1). On day-470 (1-week pre-4th-dose), the gMean-NT_50_ had remarkably decreased down to 390, by around 19% of the peak value of day-300 (2-weeks post-3rd-dose/Fig. 2A). However, by day-490 (2-weeks post-4th-dose), neutralization activity increased to 1820 (Fig. 2A). On day-550 (10-weeks post-4th-dose) and day-630 (1-week pre-5th-dose), the gMean-NT_50_ decreased down to 1121 and 477, around 60% and 15% and of the peak value of day-490 (2-weeks post-4th-dose/Fig. 2A). However, by day-650 (2-weeks post-5th-dose, bivalent), neutralization activity again increased to 1966, and was higher than the peak value of day-490 with no statistical significance (2-weeks post-4th-dose, *p* = 0.8199/Fig. 2A). Fold-changes of gMean-NT_50_ against Wuhan between pre/post-5th dose was similar with those between pre/post-4th dose (4.1 and 4.7, respectively, *p* = 0.6637/Fig. 2A). We also examined the profile of SARS-CoV-2 Spike S1-binding IgG levels of sera at six different time-points, on pre-/post-3rd, 4th, and 5th-doses (Table 2), showing that gMean S1-binding IgG level of sera taken 2-weeks post-5th-^bv^BNT dose (4688 BAU/ml; ranges 846–13,334) was similar with those of post-3rd-^mv^BNT dose (4788 BAU/ml; ranges 1344–16,634) and lower than those of post-4th-^mv^BNT dose sera with no statistical significance (6305 BAU/ml; ranges 1611–19,236; *p* = 0.1860/Table 2).

When we evaluated neutralization activity in sera pre-/post-4th-dose of ^mv^BNT vaccine using infectious Omicron variants, gMean-NT_50_ values against BA.5 on day-470 (1-week pre-4th-dose) and day-490 (2-weeks post-4th-dose) sera were 23 and 134, respectively (Fig. 2B). On day-550 (10-weeks post-4th-dose) and day-630 (1-week pre-5th-dose), the gMean-NT_50_ continuously decreased down to 63 and 44 after the 4th-dose. However, by day-650 (2-weeks post-5th-dose, bivalent), gMean-NT_50_ values against BA.5 was significantly elevated to 429 (ranges; 36–8184), 3.2-fold and 9.8-fold increases from the peak values of day-490 (2-weeks post-4th-dose, monovalent; *p* = 0.0021) and day-630 (1-week pre-5th-dose; *p* < 0.0001/Fig. 2B). Fold-changes of gMean-NT_50_ against BA.5 between pre/post-5th dose was higher than those between pre/post-4th dose with no statistical significance (9.8 and 5.8, respectively, *p* = 0.1032/Fig. 2B).

Similar profiles were observed when we examined neutralization activity against BA.2: gMean-NT_50_ values against BA.2 on day-650 (= 396; 2-weeks post-5th-dose, bivalent) was 1.6-fold higher than the peak value of day-490, but with no statistical significance (= 255; 2-weeks post-4th-dose, monovalent; *p* = 0.1698/Fig. 2B). We also evaluated neutralization activity against BQ.1.1 and XBB of day-650 (2-weeks post-5th-dose, bivalent) sera. gMean-NT_50_ values were 126 (ranges; < 20–513) and 114 (ranges; < 20–936) against BQ.1.1 and XBB, respectively, and were lower than those against BA.5 (*p* = 0.0008 and 0.0007, respectively) and against BA.2 (*p* = 0.0006 and 0.0006, respectively/Fig. 2B). Against XBB.1.5, day-650 sera showed the lowest gMean-NT_50_ values of 63 (ranges; < 20–433) among variants we tested (Fig. 2B).

### Effects of Omicron BA.4/5-adapted BNT162b2 (^bv^BNT) vaccination in sera obtained from HCWs without risk factor.

Next, we examined SARS-CoV-2 neutralization activity of sera obtained from 90 out of 225 HCWs who were younger than 60 years of age and free from pre-existing diseases/risk factors. Results of samples obtained from swab PCR- or serum N-IgG-positive participants in this cohort were also excluded.

**Figure 3:**
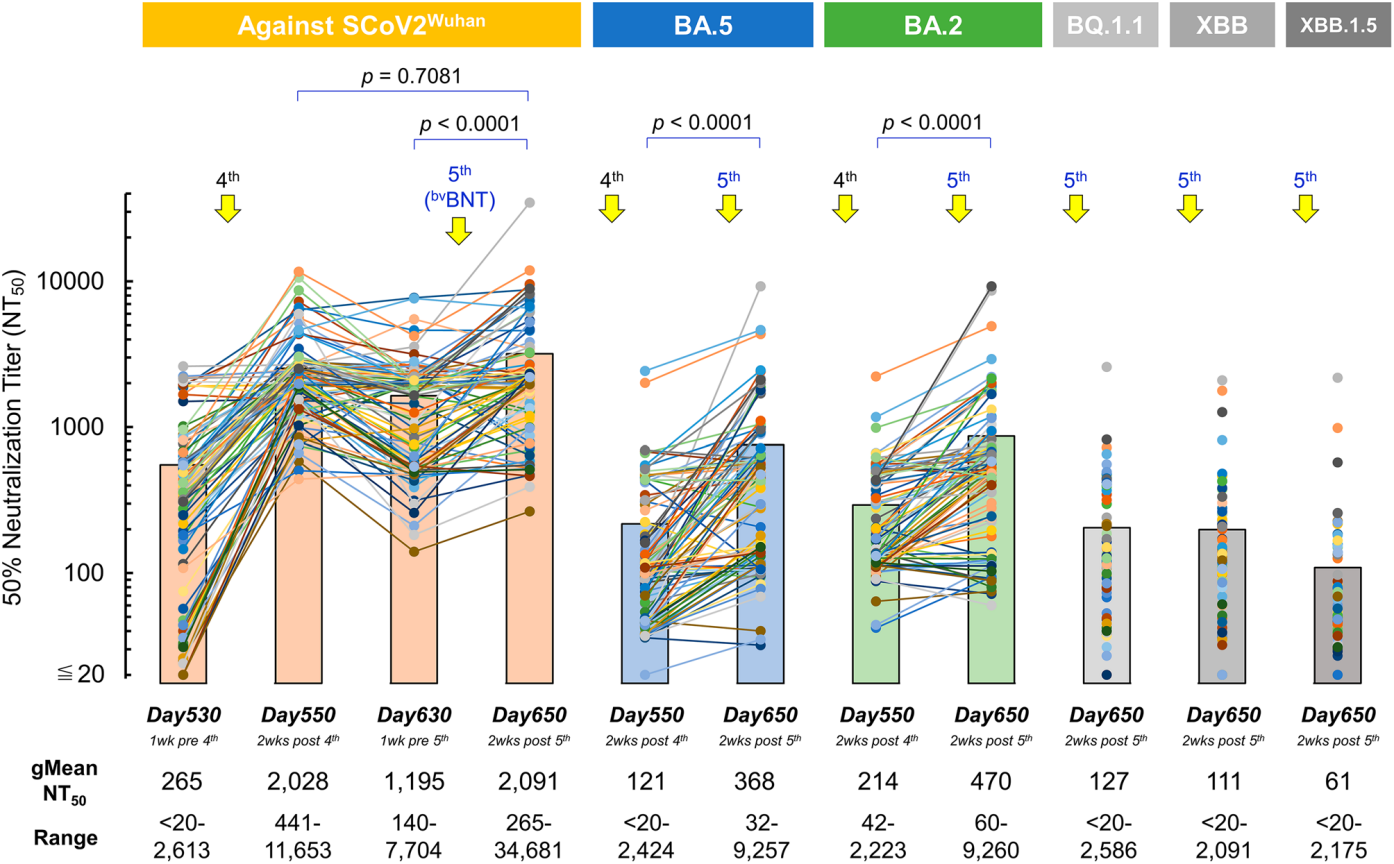
Effect of ^bv^BNT vaccination in sera obtained from HCWs without risk factor. Temporal changes of neutralizing activity of sera obtained from HCWs without risk factor over 650 days post-1st dose of ^mv^BNT are shown. 4th ^mv^BNT and 5th ^bv^BNT doses were administered on days 537 and 637, respectively (n = 90). NT_50_ of participants’ sera against infection by SCoV2^Wuhan^ and Omicrons BA.2, BA.5, BQ.1.1, XBB, and XBB.1.5 were determined using VeroE6^TMPRSS2^ cell-based neutralization assay. Solid circles denote NT_50_ titers of each participant’s serum and filled bars denote average NT_50_ titers of 90 participants’ sera at each time point. gMean-NT_50_ and ranges of NT_50_ at each time point are shown at the bottom. The circles and lines in same color indicate that the data were obtained from same participant’s sera.

Against SCoV2^wuhan^, the gMean-NT_50_ value of day-530 (1-week pre-4th-dose) was 265 (ranges; < 20–2613), and on day-550 (2-weeks post-4th-dose), the value elevated to 2028 (ranges; 441–11,653/Fig. 3). After ~ 40% decrease of NT_50_ on day-630 (1195; 1-week pre-5th-dose) from the peak value of 4th-dose, day-650 (2-weeks post-5th-dose, bivalent) sera again had an elevated value up to 2091 (ranges; 265–34,681/Fig. 3), and the values were comparable (*p* = 0.7081) to the values of day-550 (2-weeks post-4th-dose, ^mv^BNT/Fig. 3). Similar profiles were seen in sera obtained from HCWs with risk factor (Fig. 2A). Regarding fold-changes of gMean-NT_50_ against Wuhan, HCWs with risk factors’ sera between pre/post-5th dose was significantly higher than those of HCWs without risk factor (4.1 and 1.8, respectively, *p* = 0.001/Figs. 2A and 3).

When we examined the profile of S1-binding IgG (S1-IgG) levels of sera from HCWs without risk factor, S1-binding IgG of 2-weeks post-5th-^bv^BNT dose sera (4691 BAU/ml; ranges 1313–38,324) was lower than those of post-4th-^mv^BNT doses sera with statistical significance (6024 BAU/ml; ranges 1912–43,502; *p* = 0.0038/Table 2). When we compared fold-changes of pre/post 4th dose S1-IgG and pre/post 5th dose S1-IgG levels, fold-changes of pre/post 4th dose S1-IgG were significantly higher than those of pre/post 5th dose (*p* values were 0.0116 and < 0.0001, for HCWs with risk factors and HCWs without risk factor, respectively/Table 2). Also, when we compared fold-changes of S1-IgG levels in sera between HCWs with risk factors and HCWs without risk factor, fold-changes of S1-IgG levels for HCWs with risk factors pre/post 5th dose were significantly higher than those of HCWs without risk factor (*p* < 0.0001/Table 2), but no significant difference was observed in the fold-changes of S1-IgG levels pre/post 4th dose sera between HCWs with risk factors and HCWs without risk factor (*p* = 0.1029).

When we examined neutralization activity against BA.5 using sera from day-550 (2-weeks post-4th-dose) and day-650 (2-weeks post-5th-dose, bivalent), the day-650 sera showed the gMean-NT_50_ value of 368 (ranges; 32–9257), 3.0-fold higher value compared to that of day-550 sera (= 121/ranges; < 20–2424/Fig. 3). Also, against BA.2, day-650 sera showed gMean-NT_50_ value of 470 (ranges; 60–9260), 2.2-fold higher than that of day-550 sera (= 214/ranges; 42–2223/Fig. 3). When we evaluated NT_50_ against BQ.1.1 and XBB of day-650 sera, gMean-NT_50_ values were 127 (ranges; < 20–2586) and 111 (ranges; < 20–2091), respectively (Fig. 3). On the other hand, day-650 sera showed gMean-NT_50_ values of 61 (ranges; < 20–2175) against XBB.1.5 (Fig. 3).

### Effects of ^bv^BNT in sera from HCWs who had experienced symptomatic/asymptomatic breakthrough infection during Omicron wave

**Figure 4:**
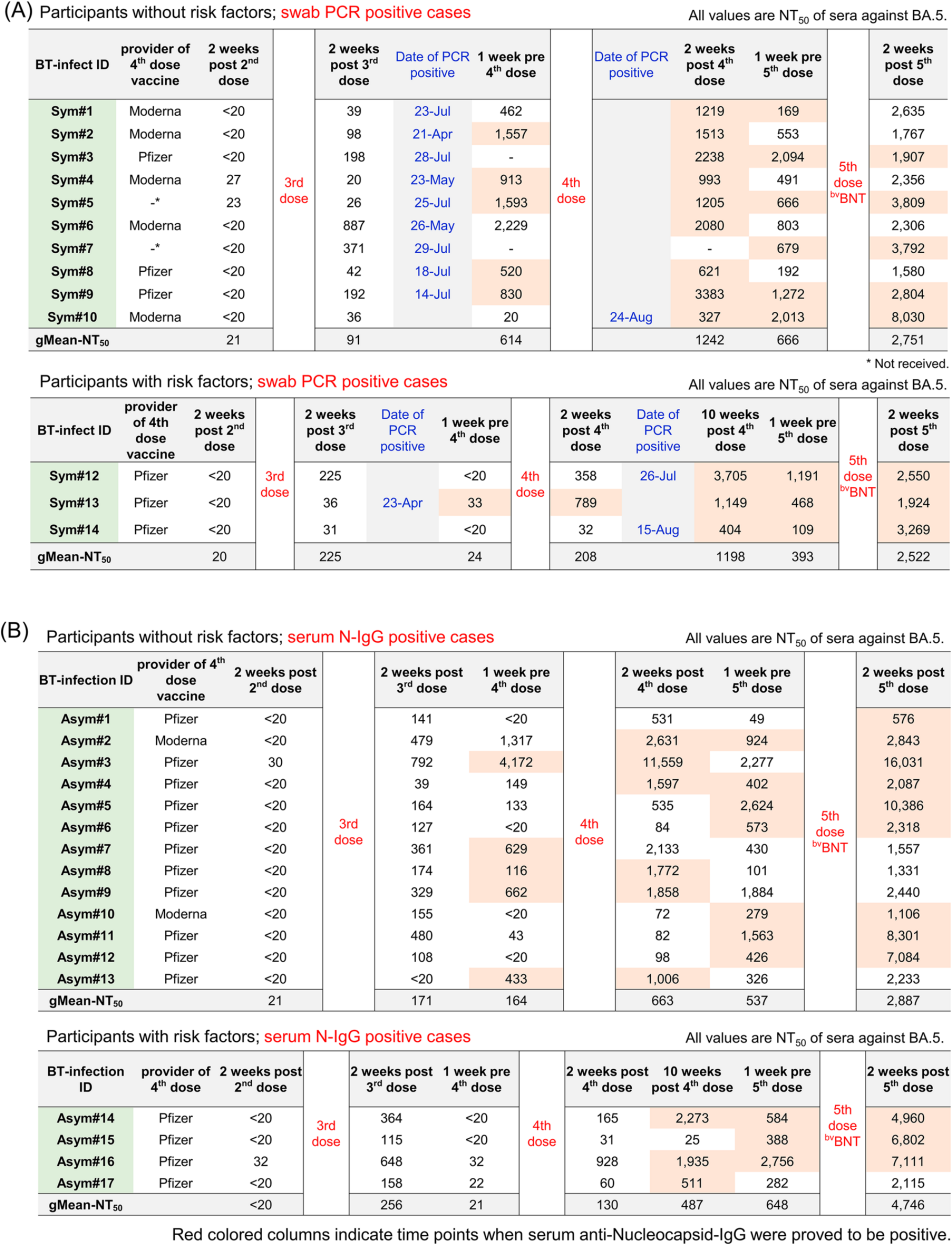
Neutralization activity against Omicron BA.5 of sera obtained from HCWs who had received booster dose of ^mv^BNT and experienced breakthrough infection during Omicron wave. Detailed information of neutralization activity (NT_50_) of sera obtained from BT-infection experienced participants against Omicron BA.5, dates of swab PCR-positive, and positive periods of serum SARS-CoV-2 nucleocapsid-specific IgG (red colored columns) are shown. (**A**) shows the results of participants with risk factors, and (**B**) shows the results of participants without risk factor.

Among HCWs enrolled in the present study, 20 participants proved to be SARS-CoV-2 positive by swab PCR from April 2022 to August 2022, and 13 out of the 20 participants received the 5th-dose ^bv^BNT vaccination after recovery. These 13 participants who had experienced symptomatic breakthrough (BT) infection and 5th-dose of vaccine were termed as “BT-Sym#1–13” (symptoms of each participant were indicated in Supplemental Table S2). Other 17 participants who had neither tested nor received any positive results for swab-PCR or antigen tests but proved to be positive for anti-SARS-CoV-2 nucleocapsid-specific-IgG in their sera obtained from August 2022 to December 2022. These 17 participants all received 5th-dose ^bv^BNT vaccine and were termed as “BT-Asym#1–17”. Detailed information regarding infection date (PCR positivity/serum N-IgG positivity) and longitudinal changes of serum NT_50_ values against Omicron BA.5 are summarized in Fig. 4 (PCR positive cases [A]; serum N-IgG positive cases [B]).

**Figure 5:**
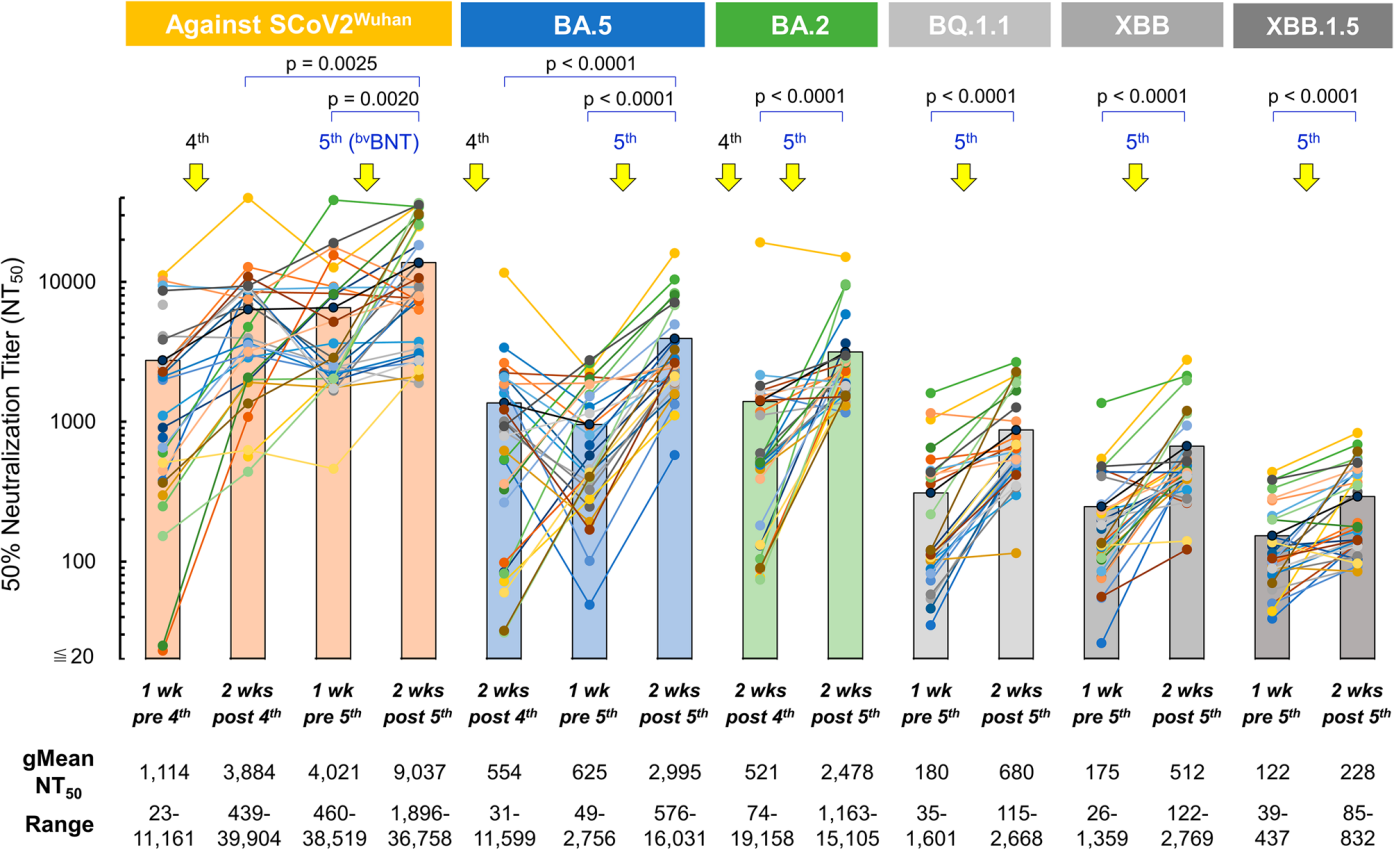
Effect of ^bv^BNT vaccination in sera from HCWs who had experienced breakthrough (BT) infection during Omicron wave. Temporal changes of neutralizing activity of sera obtained from HCWs who had experienced breakthrough (BT) infection over 650 days post-1st dose of BNT162b2 are shown (n = 30). NT_50_ of participants’ sera against infection by SCoV2^Wuhan^ and Omicrons BA.2, BA.5, BQ.1.1, XBB, and XBB.1.5 were determined using VeroE6^TMPRSS2^ cell-based neutralization assay. Solid circles denote NT_50_ titers of each participant’s serum and filled bars denote average NT_50_ titers of 30 BT-infected participants’ sera at each time point. gMean-NT_50_ and ranges of NT_50_ at each time point are shown at the bottom. The circles and lines in same color indicate that the data were obtained from same participant’s sera.

As shown in Fig. 5, the 4th-dose ^mv^BNT vaccination enhanced neutralization activity of sera against SCoV2^Wuhan^ strain by around 3.5 folds [Fig. 5/gMean-NT_50_s; from 1114 (pre-4th-dose) to 3884 (post-4th-dose)], when ~ 43% (13 out of 30) BT-infected participants had not yet been SARS-CoVf-2-positive by that time (Fig. 4). At 1-week pre-^bv^BNT 5th-dose, NT_50_ value against SCoV2^Wuhan^ strain remained high (Fig. 5/gMean-NT_50_ = 4021/ranges; 460–38,519), and 2-weeks post-^bv^BNT 5th-dose, NT_50_ value significantly increased up to 9037 (Fig. 5/ranges; 1896–36,758), 4.3-–4.6-fold higher than those of uninfected participants’ sera after 5th-dose ^bv^BNT (Figs. 2, 3).

When we examined neutralization activity against BA.5 using sera of post-4th-dose and pre-5th-dose, they showed NT_50_ values of 554 and 625, respectively (Fig. 5), slightly greater than the peak-values of uninfected participants’ post-5th-dose sera (Figs. 2, 3). Surprisingly, post-5th-dose sera of BT-infected participants showed significantly high gMean-NT_50_ value of 2995 against BA.5 (Fig. 5/ranges; 576–16,031), which was 7.0–8.1-fold greater compared to the peak-values of uninfected participants’ sera post-5th-dose (Figs. 2, 3). Also, against BA.2, post-5th-dose sera showed good gMean-NT_50_ value of 2478 (ranges; 1163–15,105), which was 4.8-fold higher value compared to that of post-4th-dose sera (= 521/ranges; 74–19,158/Fig. 5), and was 5.1–6.3 folds greater value compared to those of uninfected participants’ sera post-5th-dose (Figs. 2, 3).

When we evaluated NT_50_ against BQ.1.1 using pre- and post-5th-dose sera of previously BT-infected participants, gMean-NT_50_ values were 180 (ranges; 35–1601) and 680 (ranges; 115–2668), respectively (Fig. 5), which showed 3.8-fold enhancement following the ^bv^BNT vaccination. These data perhaps show the cross-neutralization elicited by the 5th-dose ^bv^BNT between against BA.5 and against BQ.1.1 considering that BQ.1.1 emerged from BA.5^13^.

Against XBB, gMean-NT_50_ values of pre- and post-5th-dose sera were 175 (ranges; 26–1601) and 512 (ranges; 122–2668) respectively (Fig. 5), showing 2.9-fold enhancement of neutralization activity after the 5th-^bv^BNT dose. Also, NT_50_ values against BQ.1.1 and XBB of BT-infected participants’ sera post-5th-dose were ~ 5.4- and ~ 4.5-fold higher than those of uninfected participants’ sera post-5th-dose (Figs. 2, 3). When we evaluated NT_50_ against XBB.1.5 using post-5th-dose sera of BT-infected participants, gMean-NT_50_ values were 228 (ranges; 85–832/Fig. 5). %Reduction of gMean-NT_50_ values of post 5th-dose sera against BQ.1.1, XBB, and XBB.1.5 compared with those against vaccine-strains (Wuhan and BA.5) are summarized in Table S3.

## Discussion

In the present cohort study, we studied in detail the effectiveness of BA.4/5 adapted bivalent BNT162b2 (^bv^BNT) vaccine using various infectious SARS-CoV-2 s, by examining the participants’ sera obtained pre-/post-2nd–4th-doses of ^mv^BNT, from participants with/without risk factors or who had experienced BT-infection during the Omicron-wave (January 2022 through December 2022) in Japan.

In our previous data with ^mv^BNT vaccinations between post-3rd and 4th-doses, neutralization activity against SCoV2^Wuhan^ elicited by 4th-dose of ^mv^BNT were not greater than those after 3rd-dose of ^mv^BNT^17, 18^ (Supplemental Table S1). Similarly, the magnitudes of neutralizing activity against SCoV2^Wuhan^ after the 5th-^bv^BNT dose were not greater compared to the significantly boosted response elicited by the 3rd-^mv^BNT in participants with/without risk factors (Supplemental Table S1). These limited restoration regarding neutralization activity against SCoV2^Wuhan^ by 5th-^bv^BNT seems to reflect the difference the amounts of mRNA containing against original SCoV2^Wuhan^ between 3rd-dose and 5th-dose (30 μg of mRNA for 3rd-dose of ^mv^BNT, and 15 μg of mRNA for 5th-dose of ^mv^BNT). However, 4th dose-^mv^BNT also contains 30 μg of mRNA against original SCoV2^Wuhan^, but 5th-^bv^BNT elicited higher neutralization activity against SCoV2^Wuhan^ than those of 4th-^mv^BNT (Supplemental Table S1), indicating ^bv^BNT may have different property from that of ^mv^BNT against SCoV2^Wuhan^.

We also evaluated sera post-5th-^bv^BNT dose against not only BA.5 but also BA.2. All the post-5th-dose sera examined in the current study demonstrated significantly more robust neutralization activity against BA.5 and showed favorable neutralization activity also against BA.2 (p < 0.0001 for both BA.5 and BA.2 in Figs. 2 and 3).

In the present study, we also focused on the groups of participants who experienced BT-infection during the Omicron wave period (Fig. 4 and 5). BT-infected participants showed significant enhancement of neutralization activity after ^bv^BNT dose against SCoV2^Wuhan^, BA.2, and BA.5, as well as BQ.1.1 and XBB. These results suggest that repeated stimulation caused by the exposure to Omicron’s Spike protein elicited broad and stronger neutralization activity against multiple SARS-CoV-2 variants. If it is the case and if further infection waves by SARS-CoV-2 variants arrive, booster ^bv^BNT doses may have to be considered, although further data on the range of neutralization elicited by ^bv^BNT have to be carefully examined.

When we compared gMean-S1-binding IgG levels and gMean-NT_50_ against BA.5 in sera obtained 2 weeks-post 3rd-^mv^BNT dose between symptomatic BT-infection (Sym-BTI) group and asymptomatic BT-infection (Asym-BTI) group, significant difference was observed in S1-binding IgG levels. Sym-BTI group showed gMean-S1-binding IgG of 3834 (ranges 1401–9843), while Asym-BTI group showed 1.9-fold higher gMean-S1-binding IgG value of 7272 (ranges 2668–15,963; *p* = 0.007/Table S4). Similarly, post 3rd-dose of Asym-BTI group’s sera showed 2.3-fold higher gMean-NT_50_ value against BA.5 compared to those of Sym-BTI group with no statistical significance (191.4 and 83.4, respectively; *p* = 0.0523/Table S4). These results might be an explanation why these groups showed different disease severity after SARS-CoV-2 BT-infection post 3rd-dose of original ^mv^BNT vaccination.

The present data show that the 5th-^bv^BNT dose elicits greater levels of SARS-CoV-2-neutralizing activities against various SARS-CoV-2 variants including Omicron sublineages such as BA.2 and BA.5 although elicitation of neutralization against BQ.1.1, XBB, and XBB.1.5 is limited, indicating that more improved anti-SARS-CoV-2 vaccines capable of eliciting further broader and stronger neutralization are required to further better respond to the current COVID-19 pandemic. It was also suggested that individuals who previously experienced SARS-CoV-2 infection (mostly with Omicron variants) may have more robust neutralization against Omicron variants, which endorses vaccination with ^bv^BNT dose following vaccination with ^mv^BNT. However, further evaluation should be required for the administration of booster bivalent mRNA vaccination to the individuals who experienced recent BT-infection.

## Methods

### Participants and serum specimens.

The vaccination (on days 0, 21, 287, and 537, 30 μg of mRNA/each dose for ^mv^BNT, and on day 637, 15 μg of mRNA against original strain and 15 μg of mRNA for ^bv^BNT) and serum collection (on day-7, -28, -60, -90, -150, -280, -300, -360, -470, -490, -530, -550, -630, and -650 post-1st-dose) were carried out. Samples were collected from vaccinated health care workers at Japan Community Health Care Organization (JCHO), Kumamoto General Hospital (Kumamoto, Japan). In this report, 23 participants had previously received 4th-dose of monovalent BNT162b2 vaccine (^mv^BNT) 2 months earlier than other participants since they had a risk(s) of developing severe COVID-19. In contrast, 90 participants who were younger than 60 years of age and free from pre-existing diseases/risk factors received 4th-dose of ^mv^BNT vaccination 2 months later than participants with risk factors, so that the interval between the 4th-dose and 5th-dose were 2 months shorter than participants with risk factors (See Fig. 1).

Samples were analyzed at Kumamoto University in Kumamoto and the National Center for Global Health and Medicine (NCGM) in Tokyo. The Ethics Committee from the Kumamoto General Hospital, NCGM, and Kumamoto university approved this study (Kumamoto General Hospital No. 180, NCGM-G-004176-00, and Kumamoto university No 2643). Each participant provided a written informed consent, and this study abided by the Declaration of Helsinki principles. The infection by a series of Omicron variants was dominant in Japan largely from January, 2022 through December, 2022. We defined the period of SARS-CoV-2 infection in Japan as “Omicron wave period”.

### Cells and viruses

VeroE6^TMPRSS2^ cells^19^ were obtained from Japanese Collection of Research Bioresources (JCRB) Cell Bank (Osaka, Japan). VeroE6^TMPRSS2^ cells were maintained in DMEM supplemented with 10% FCS, 100 µg/ml of penicillin, 100 µg/ml of streptomycin, and 1 mg/ml of G418.

SARS-CoV-2 NCGM-05-2N strain (SCoV2^05-2N^) was isolated from nasopharyngeal swabs of a patient with COVID-19, who was admitted to the NCGM hospital^20^. hCoV-19/Japan/TKYS02037/2022 (Omicron/BA.2; SARS-CoV-2^2037^, GISAID Accession ID; EPI_ISL_9397331), hCoV-19/Japan/TKYS14631/2022 (Omicron/BA.5; SARS-CoV-2^TKYS14631^, GISAID Accession ID: EPI_ISL_12812500.1), hCoV-19/Japan/TY41-796/2022 (BQ.1.1; SARS-CoV-2^TY41-796^, GISAID Accession ID: EPI_ISL_15579783), and hCoV-19/Japan/TY41-795/2022 (XBB; SARS-CoV-2^TY41-795^, GISAID Accession ID: EPI_ISL_15669344) were provided from Tokyo Metropolitan Institute of public Health, Japan. hCoV-19/Japan/23-018-P1/2022 (XBB.1.5; SARS-CoV-2^23-018^, GISAID Accession ID: EPI_ISL_16889601) was provided by National Institute of Infectious Diseases, Japan. Each variant was confirmed to contain each variant-specific amino acid substitutions.

### Neutralization assay procedure.

The neutralizing activity of sera from vaccinated individuals was determined by quantifying the serum-mediated viral suppression in SARS-CoV-2-infected VeroE6^TMPRSS2^ cells as previously described with minor modifications^14, 21^. In brief, each serum was serially diluted in culture medium. The diluted sera were incubated with 100 TCID_50_ of viruses at 37 °C for 20 min (final serum dilutions were 1:20, 1:62.5, 1:250, 1:600, 1;1,000, 1:4000, 1:16,000, and 1:64,000), after which the serum-virus mixtures were inoculated to VeroE6^TMPRSS2^ cells (1.0 × 10^4^/well) in 96-well microtiter culture plates. SARS-CoV-2 strains used in this assay were as follows: a wild-type Wuhan strain SCoV2^05-2N^, Omicron strains SARS-CoV-2^2037^ (BA.2; contains K417N/T478K/E484A/N501Y/D614G mutations in Spike), SARS-CoV-2^TKYS14631^ (BA.5; contains K417N/L452R/T478K/E484A/F486V/N501Y/D614G mutations in Spike), SARS-CoV-2^TY41-796^ (BQ.1.1; contains R346T/K417N/K444T/L452R/N460K/T478K/E484A/F486V/N501Y/D614G.

mutations in Spike), SARS-CoV-2^TY41-795^ (XBB; contains R346T/K417N/T478K/V445P/G446S/N460K/E484A/F486S/N501Y/D614G mutations in Spike), and SARS-CoV-2^23-018^ (XBB.1.5; contains R346T/K417N/T478K/V445P/G446S/N460K/E484A/F486P/N501Y/D614G mutations in Spike). After culturing the cells for 3 days, the levels of virally caused cytopathic effect (CPE) observed in SARS-CoV-2-exposed cells were determined using the WST-8 assay employing Cell Counting Kit-8 (Dojindo, Kumamoto, Japan). The serum dilution that gave 50% inhibition of CPE was defined as 50% neutralization titer (NT_50_s). Each serum was tested in duplicates. All *p* values presented in the Figures and Results were calculated using the t-test.

### Supplementary Information

Supplementary Tables.

## Data Availability

The data sets generated during this study are available from the corresponding author upon request.
