# Supplementary material for: Neutralization against Omicron sublineages (BA.2/BA.5/BQ.1.1/XBB/XBB.1.5) in bivalent BNT162b2-vaccinated HCWs with or without risk factors, or following BT infection with Omicron
Source: Sci Rep. 2023 Oct 13;13:17404. doi: 10.1038/s41598-023-44484-x (PMC10575932; doi:10.1038/s41598-023-44484-x)
Supplement: Supplementary file 1 — Supplementary Tables. [file 41598_2023_44484_MOESM1_ESM.pdf]

Supplemental materials

Effect of BA.4/5-adapted bivalent BNT162b2 vaccine against various Omicron sublineages (BA.2/BA.5/BQ.1.1/XBB/XBB.1.5) in sera from HCWs with/without risk factors and who had experienced BT-infection during Omicron wave.

Masayuki Amano, Sachiko Otsu, Yukari Uemura, Yasuko Ichikawa, Shota Matsumoto,

Nobuyo Higashi-Kuwata, Shuzo Matsushita, Shinya Shimada, Hiroaki Mitsuya.

Contents

Supplementary Table S1; page 2

Supplementary Table S2; page 3

Supplementary Table S3; page 4, 5

Supplementary Table S4; page 6

Table S1. Alternation profiles of gMean-NT_{50} observed in post 2nd, 3rd, 4th, and 5th-BNT162b2 doses sera against SARS-CoV-2^{Wuhan}.

Time points when sera were obtained	gMean-NT_{50} against $SCoV2^{Wuhan}$	
	HCWs with risk factors (n=23)	HCWs without risk factor (n=90)
Day-28; 2-weeks post 2nd-dose	283	416
Day-300; 2-weeks post 3rd-dose	2,009	2,540
Days490/550;<br>2-weeks post 4th-dose	1,820 (day490)	2,028 (day550)
Day650; 2-weeks post 5th-dose	1,966	2,091

Geometric mean (gMean) NT_{50} titers of the peak values post $2^{nd}/3^{rd}/4^{th}$-doses of monovalent BNT162b2 (^{mv}BNT) and 5th-dose of bivalent BNT162b2 (^{bv}BNT) of sera obtained from participants with risk factors (n=23) and participants without risk factor (n=90) against SARS-CoV-2^{Wuhan} ($SCoV2^{Wuhan}$) are indicated. NT_{50} values were determined in cell-based assays using infectious SARS-CoV-2^{Wuhan} strain and $VeroE6^{TMPRSS2}$ cells.

Table S2. Symptoms of each symptomatic BT-infected participant.

BT-infection ID	Days of symptoms continued (within 10 days from onset)							
	Fever	General fatigue	Sore throat	Cough	Runny nose	Headache	Digestive symptoms	Others
Sym#1	1	1	3	0	1	1	0	-
Sym#2	0	2	0	0	0	0	0	-
Sym#3	5	10	9	10	0	6	0	-
Sym#4	6	6	4	6	0	0	0	Lumbago dysgeusia
Sym#5	6	8	8	10	4	8	5	-
Sym#7	4	7	6	10	1	4	3	-
Sym#8	10	8	6	6	10	10	0	dysgeusia
Sym#10	2	10	6	5	4	3	0	-
Sym#11	1	3	3	0	0	0	0	-
Sym#13	3	5	3	4	5	5	0	-

Symptoms observed in each symptomatic BT-infected participant within 10 days from onset are shown.

Table S3. %Reduction of geometric mean NT_{50} values of post 5th dose sera against BQ.1.1, XBB, and XBB.1.5 compared to those against vaccine-strains.

A) HCWs with risk factors

Strain	Wuhan	BQ.1.1	XBB	XBB.1.5
gMean NT_{50}	1,966	126	114	63
%reduction from Wuhan	-	93.6	94.2	96.8
p value	-	<0.0001	<0.0001	<0.0001
Strain	BA.5	BQ.1.1	XBB	XBB.1.5
gMean NT_{50}	429	126	114	63
%reduction from BA.5	-	70.6	73.4	85.3
p value	-	0.0008	0.0007	<0.0001

B) HCWs without risk factor

Strain	Wuhan	BQ.1.1	XBB	XBB.1.5
gMean NT_{50}	2,091	127	111	61
%reduction from Wuhan	-	93.9	94.7	97.1
p value	-	<0.0001	<0.0001	<0.0001
Strain	BA.5	BQ.1.1	XBB	XBB.1.5
gMean NT_{50}	368	127	111	61
%reduction from BA.5	-	65.5	69.8	83.4
p value	-	<0.0001	<0.0001	<0.0001

C) HCWs who experienced BT-infection

Strain	Wuhan	BQ.1.1	XBB	XBB.1.5
gMean NT_{50}	9,037	680	512	228
%reduction from Wuhan	-	92.5	94.3	97.5
p value	-	<0.0001	<0.0001	<0.0001
Strain	BA.5	BQ.1.1	XBB	XBB.1.5
gMean NT_{50}	2,995	680	512	228
%reduction from BA.5	-	77.3	82.9	92.4
p value	-	<0.0001	<0.0001	<0.0001

%Reduction of gMean-NT_{50} values of 2-weeks post ^{bv}BNT sera against BQ.1.1, XBB, and XBB.1.5 compared to those against vaccine-strains (Wuhan and BA.5). **A**, **B**, and **C** represented results of sera obtained from HCWs with risk factors, HCWs without risk factor, and BT-infection experienced HCWs, respectively. All *p* values presented were calculated using the t-test.

Table S4. Comparison of gMean-S1-binding IgG levels and gMean-NT_{50} against BA.5 in sera obtained 2 weeks-post 3rd-^{mv}BNT dose between symptomatic BT-infection group and asymptomatic BT-infection group.

	Day300; 2-weeks post 3rd-dose sera	
	Symptomatic BT-infection (n=13)	Asymptomatic BT-infection (n=17)
gMean-S1-binding IgG (BAU/ml)	3,834 (ranges; 1,401-9,843)	7,272 (ranges; 2,668-15,963)
gMean-NT_{50} against BA.5	83.4 (ranges; 20-887)	191.4 (ranges; <20-792)

Geometric mean (gMean) S1-binding IgG levels and gMean-NT_{50} titers against BA.5 of sera obtained 2-weeks post 3rd-doses of monovalent BNT162b2 (^{mv}BNT) from participants with symptomatic BT-infection (n=13) and Asymptomatic BT-infection (n=17) are indicated. NT_{50} values were determined in cell-based assays using infectious SARS-CoV-$2^{BA.5}$ variant and VeroE$6^{TMPRSS2}$ cells.
